# Plasmid selection in *Escherichia coli *using an endogenous essential gene marker

**DOI:** 10.1186/1472-6750-8-61

**Published:** 2008-08-11

**Authors:** Shan Goh, Liam Good

**Affiliations:** 1 Department of Cell and Molecular Biology, Karolinska Institute, Stockholm, SE-17177, Sweden; 2 Department of Pathology and Infectious Diseases, Royal Veterinary College, University of London, AL9 7TF, UK

## Abstract

**Background:**

Antibiotic resistance genes are widely used for selection of recombinant bacteria, but their use risks contributing to the spread of antibiotic resistance. In particular, the practice is inappropriate for some intrinsically resistant bacteria and in vaccine production, and costly for industrial scale production. Non-antibiotic systems are available, but require mutant host strains, defined media or expensive reagents. An unexplored concept is over-expression of a host essential gene to enable selection in the presence of a chemical inhibitor of the gene product. To test this idea in *E. coli*, we used the growth essential target gene *fabI *as the plasmid-borne marker and the biocide triclosan as the selective agent.

**Results:**

The new cloning vector, pFab, enabled selection by triclosan at 1 μM. Interestingly, pFab out-performed the parent pUC19-ampicillin system in cell growth, plasmid stability and plasmid yield. Also, pFab was toxic to host cells in a way that was reversed by triclosan. Therefore, pFab and triclosan are toxic when used alone but in combination they enhance growth and plasmid production through a gene-inhibitor interaction.

**Conclusion:**

The *fabI*-triclosan model system provides an alternative plasmid selection method based on essential gene over-expression, without the use of antibiotic-resistance genes and conventional antibiotics.

## Background

Antibiotic resistance marker genes are commonly used to select and maintain recombinant bacteria in the presence of antibiotics. However, the use of antibiotics is undesirable for manufacturing gene therapy products [1,2]. Also, the introduction of antibiotic resistance genes into biohazardous strains is not recommended [3], and antibiotic selection fails in bacteria that are naturally resistant [3,4]. Finally, the use of antibiotics can be costly in industrial scale production, especially in the case of enzymatic depletion of antibiotic during culture. An alternative system should avoid antibiotic resistance markers and therapeutic antibiotics, be cost effective, but still be convenient, robust and flexible.

A variety of strategies for antibiotic-free selection have been developed, but none are widely used in bacteria. The first reported non-antibiotic system involves an auxotrophic bacterial strain and complementation using a plasmid-encoded biosynthesis gene, such that only transformants grow on defined media lacking the nutrient [5]. Another system is termed repressor-titration, where the *lac *operator functions as the vector-borne selection marker, which de-represses a modified chromosomal essential gene [6]. A third general approach is to alter the expression of growth essential genes using synthetic [7] or expressed antisense sequences [8]. Unfortunately, these and other existing non-antibiotic systems suffer from a need for mutant host strains or expensive reagents, and in some cases low efficiency.

It has been observed that over-expression of a growth essential gene results in reduced susceptibility to the gene product inhibitor [9]. Here we considered a strategy based on an essential gene as the plasmid-borne marker and a specific protein level inhibitor as the selective agent. To test the idea, we chose *fabI*-triclosan as a model combination because it is a well-studied gene-inhibitor pair: FabI (enoyl ACP reductase) catalyzes fatty acid elongation [10] and confers reduced susceptibility to triclosan when over-expressed in *E. coli *[11,12]; triclosan inhibits FabI through binding at the ACP-enoyl substrate site, forming a stable FabI/NAD+/triclosan ternary complex [13]. In addition, triclosan is a biocide that fulfils the criteria of a non-antibiotic [14]. It is stable, easy to handle, inexpensive and approved for use in many hygiene, household and industrial applications [15,16].

Although triclosan is not used as a systemic therapeutic, it is important to consider possible risks associated with new applications. Most importantly, there is concern that triclosan use may contribute to antibiotic resistance [17]; indeed, resistant mutants can be generated in the laboratory [18-20]. However, studies of bacteria in non-laboratory conditions exposed to biocide-concentrations of triclosan did not find a correlation between antibiotic resistance and decreased triclosan susceptibility [21-23]. Regulatory agencies continue to approve the use of triclosan in domestic and clinical products [24,25], and it appears to be a comparatively safe choice as a selection agent.

In this study, we tested *fabI*-marker plasmid selection by triclosan and observed efficient and stable selection. The new system out-performed the parent antibiotic system in growth and plasmid production in the presence of triclosan. Cells containing the *fabI*-marker plasmid displayed toxic effects in the absence of triclosan, suggesting an "addictive" effect, which may aid plasmid containment.

## Methods

### Bacterial strains, plasmids and media

The *E. coli *strains used in this study were DH5α (Invitrogen), XL1-Blue (Strategene), HB101, BL21 (Strategene) and K12 (Coli Genetics Stock Center, Yale U.). Plasmids were pUC19 (New England Biolabs), pBAD18 and pBAD18s (National Institute of Genetics, Japan). Media were SOC and LB (GIBCOBRL) supplemented with ampicillin (LBA, 100 μg/ml ampicillin, (Sigma)), triclosan (LBT, 1 μM triclosan; Ciba) and arabinose (Sigma). Triclosan was used as a 1 M stock in DMSO.

### Construction of pFab, pUCFA and pBFab

An *Eag*I site was created at nucleotide position 1621 of pUC19 (New England Biolabs), immediately downstream of *bla*, by PCR with primers (5'cgtcggccgttaccaatgcttaatcag and 5'cgccggccggaccaagtttactcatat). The amplicon was digested with *Eag*I (New England Biolabs), ligated with T4 DNA ligase and transformed into DH5α for propagation. To construct pFab, the *bla *gene was excised from pUC19 with *Ssp*I and *Eag*I, and replaced with *fabI *together with its promoter, which was amplified from K12 genomic DNA using the primers 5'ccggatatcgtgctggagaatattcg and 5'gcgcggccgttatttcagttcgagttcgtt. The amplicon was digested with *EcoR*V and *Eag*I and used to create the pFab vector. *E. coli *DH5α were transformed with pFab and plated onto LB with 0.5 – 5 μM triclosan. Transformants were subsequently maintained in 1 μM triclosan (LBT). The *fab*I gene was also cloned into pUC19 at *Sph*I and *Bam*HI within the MCS (pUCFA) in a similar way using the primers 5'ccggcatgcgtgctggagaatattcg and 5'ccggatccgattatttcagttcgagt. Competent *E. coli *DH5α were transformed with pUCFA and plated onto LBA and LBT.

To induce expression of *fabI *from P_BAD_, the *fabI *amplicon generated from primers (5'cggaattcgaatgggttttctttccgg and 5'cctctagagattatttcagttcgagt) was digested with *Eco*RI and *Xba*I (New England Biolabs) and cloned into pBAD18s, which was similarly digested, to yield pBFab1. Expression of *fabI *in pBAD18 required a Shine Dalgarno sequence, which was predicted to be uaagga at position -13 relative to the start codon. Primers (5'cggaattctcaacaataaggattaaagc and 5'cctctagagattatttcagttcgagt) were used for amplification of *fabI *with its Shine Dalgarno sequence, and cloned into pBAD18 to yield pBFab6. *E. coli *DH5α were transformed with the pBFab plasmids and plated onto LBA, LBT and LBT with 0.2% (w/v) arabinose.

### Plasmid and transformant properties

Transformation efficiencies of chemically competent DH5α cells were determined as recommended by the manufacturer (Invitrogen).

Plasmid yields were determined from five clones of pUC19 and pFab. Plasmids were isolated from one ml overnight cultures grown under selection using a miniprep kit (Qiagen) and quantified by OD_260 _readings. Plasmids (50 ng) were digested with *Bam*HI and electrophoresed in a 1% agarose gel.

Plasmid stability was assayed either in the presence or absence of selection. Overnight cultures grown under selection at 37°C with shaking were diluted 1000 × in 5 ml LB with or without selection, and aliquots of the time zero cultures were diluted and plated onto LB plates containing X-Gal (20 μg/ml, Saveen). The time zero cultures were grown and diluted as above at 24 and 48 h, and aliquots of the 48 h cultures were plated. The ratios of blue colonies to total colonies on LB plates with X-Gal (20 μg/ml) were determined at 0 h and 48 h, from which % plasmid-bearing cells at 48 h were calculated. Five independent clones of pUC19 and pFab were used to provide replicates.

Plasmid abundance was determined in two ways. First, to compare band intensities of genomic (gDNA) to plasmid DNA (pDNA) in an agarose gel, total genomic DNA was extracted from five different clones of pUC19 and pFab clones grown under selection for 16 h. Five cultures of K12, derived from five single colonies, were grown in LB for DNA extraction using the Bacterial GenElute system (Sigma) for one ml of overnight culture. Total DNA (10 μl) was electrophoresed in a 1% agarose gel, stained with ethidium bromide and scanned using a Typhoon 9400 (Amersham Biosciences). Band intensities were determined by using ImageQuant software (Amersham Biosciences). Second, relative quantitative PCR (qPCR) was carried out by using the plasmid *lacZα *gene as the target gene and single copy chromosomal *dxs *as the reference gene [26]. K12 gDNA containing a single copy of *lacZα *and *dxs *was used as a calibrator. Primers amplifying the target gene (5'gtgctgcaaggcgattaagtt and 5' cactggccgtcgttttacaa), and reference gene (5'cgagaaactggcgatcctta and 5'cttcatcaagcggtttcaca) were validated for similar amplification efficiencies. Real time data analyses were carried out by the 2^-ΔΔCT ^method for relative qPCR [27]. Total DNA concentrations were determined by OD_260 _absorbance for qPCR. Each 25 μl of PCR reaction contained 12.5 μl of SYBR Green PCR buffer (Eurogentec), 100 nM of each primer (Biomers) and 5 ng total DNA.

Growth rates were calculated from the exponential phase of growth [28], which was monitored as increased OD_550 _over time by the VERSAmax spectrophotometer (Molecular Devices). An overnight culture (16 h), standardized by OD_550 _to yield approximately 7 × 10^5 ^cfu/ml, was grown in 200 μl volumes per well in a 96-well plate for 24 h with agitation for 5 s every 5 min, when OD_550 _readings were taken. Triclosan was added to give 0 – 2 μM Triclosan and 1% DMSO final concentration.

The host range of pFab within commonly used *E. coli *cloning strains was tested by transformation of XL1-Blue (Stratagene), HB101 and BL21, followed by selection on LBT plates. Plasmid DNA integrity and abundance was determined by plasmid extraction, digestion of 100 ng DNA with *Bam*HI and fractionation in a 1% agarose gel.

### Inducible expression of *fabI*

Clones of pBFab1 and pBFab6 were grown in LBA for 16 h, diluted to approximately 5 – 9 × 10^6 ^cfu/ml in LBT and aliquots of 180 μl were added to wells in a 96 well plate. Arabinose was added to a final concentration of 0 – 5% and the final volume per well was made up to 200 μl with water. Clones of pBAD18 and pBAD18s were included as controls. Cultures were grown for 24 h in the VERSAmax spectrophotometer (Molecular Devices) with agitation for 5 s every 5 min, followed by an OD_550 _reading. The growth rate at each arabinose concentration was calculated as described above.

### Cell viability

DH5α/pFab were grown overnight in the absence or presence of 0.5 – 2 μM triclosan and subjected to SYTOX green staining and flow cytometry, as previously described [29]. DH5α/pUC19 was included as a control to determine staining levels of live and heat-treated dead cells. Samples were excited with a 488 nm air-cooled argon ion laser in the CyFlow SL flow cytometer (Partec GmbH). Threshold settings were enabled on forward scatter to exclude cell debris. The forward and side scatter dot plot was used to identify and gate cell populations. Fluorescence was measured at 520 nm. Viable and dead cell populations were counted using the Partec, FloMax software version 2.4e.

### Growth competition

Growth competition between DH5α and plasmid bearing cells was carried out as previously described, with modifications [30]. Overnight (24 h) cultures of DH5α, DH5α/pUC19 and DH5α/pFab were prepared in LB, LBA and LBT, respectively. Equal volumes of DH5α and plasmid bearing cultures were mixed and diluted 1:100 in 10 ml fresh LB for further growth. An aliquot of the diluted mixed culture was simultaneously plated onto selective and non-selective media for cell counts. After the mixed culture was incubated for 24 h with shaking at 37°C, it was diluted 1:100 in 10 ml of fresh LB for further growth and plated, as above. This procedure was repeated 5 times over six days. The numbers of ampicillin or triclosan resistant colonies were scored relative to the total CFUs.

## Results

### Vectors containing *fabI *enable triclosan selection

**Figure 1:**
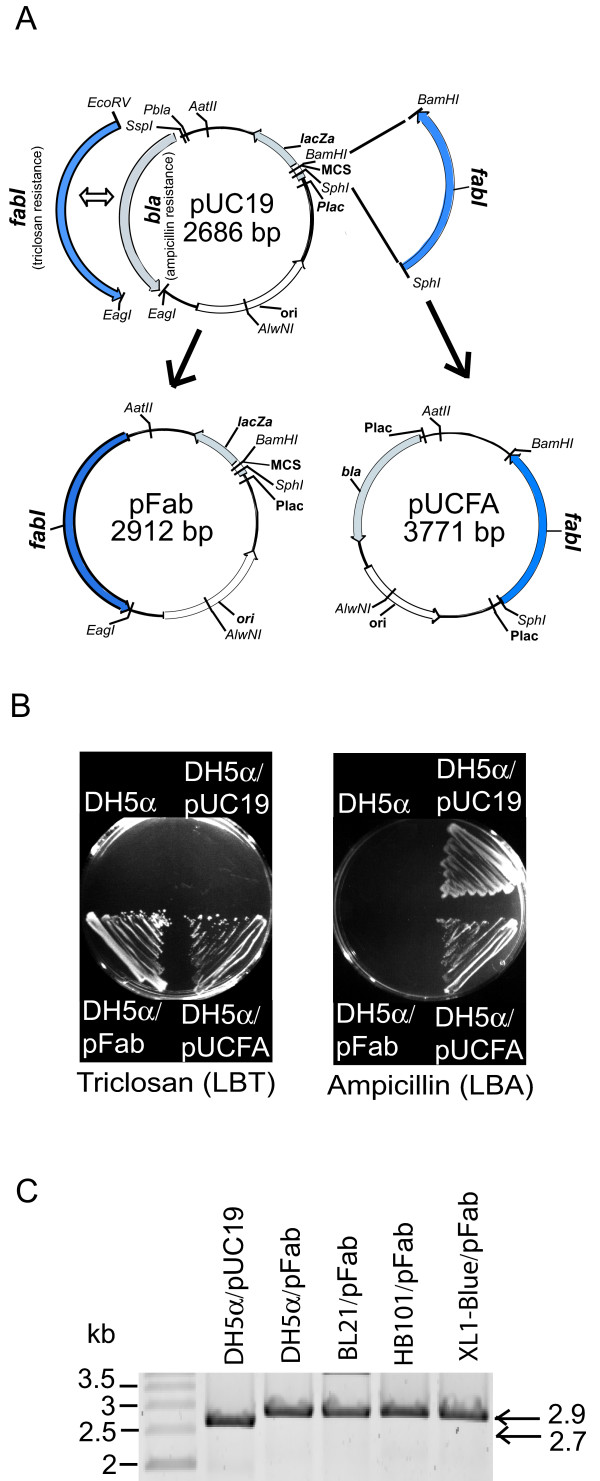
**Vector construction and triclosan selection**. (**A**) The *bla *gene in pUC19, which confers ampicillin resistance, was replaced with *fabI *and its promoter region (pFab). The pUC19 multiple cloning site (MCS) is retained, however *Hin*cII, *Hind*III and *Pst*I are not unique in pFab. The *fabI *cassette in pFab can be transferred to other pUC-derived plasmids using the *Aat*II and *AlwN*I restriction sites. The *fabI *cassette was also inserted into the MCS of pUC19 to obtain pUCFA. All plasmids are available from the authors. (**B**) Growth of pFab and pUCFA clones on LBT and LBA plates. (**C**) Plasmids propagated in different *E. coli *hosts were digested with *Bam*HI and analyzed by gel electrophoresis.

To test the potential of *fabI *as a selective marker for cloning, two vectors derived from pUC19 were constructed. First, we constructed pFab, where *fabI *together with its native promoter replaces the ampicillin resistance gene (*bla*) in pUC19. Second, to enable selection with triclosan or ampicillin, we constructed pUCFA, which contains the *fabI *cassette cloned into the pUC19 MCS (Figure 1A). *E. coli *strain DH5α was transformed with pFab, pUCFA or pUC19 and transformants were selected on LB plates containing triclosan (LBT) or ampicillin (LBA). As anticipated, *fabI *inserts enabled selection on triclosan containing plates. Colonies formed on LBT were more variable in size than colonies on LBA. However, triclosan-resistant colonies of all sizes maintained resistance (Figure 1B) and displayed uniform colony morphologies upon re-plating. In contrast to pUC19, we did not observe plasmid-free colonies or satellite colonies when using pFab and pUCFA. Therefore, the *fabI*-triclosan system enables non-antibiotic selection and maintains stable recombinant strains.

To test whether *fabI*-triclosan selection could function well in other *E. coli *strains, pFab was transformed into strains BL21, HB101 and XL1-blue. Similar to the results in DH5α, we observed efficient selection and high yield plasmid production (Figure 1C). To test whether *fabI *can enable triclosan selection in other vectors, the pFab cassette was inserted into the multiple cloning site of pGEM-3Zf and also used to replace the ampicillin resistance gene in the low copy number vector pBR322. In both cases, triclosan resistant colonies were selected (data not shown). Therefore, the *fabI*-triclosan system enables efficient selection in commonly used vectors and *E. coli *strains.

**Figure 2:**
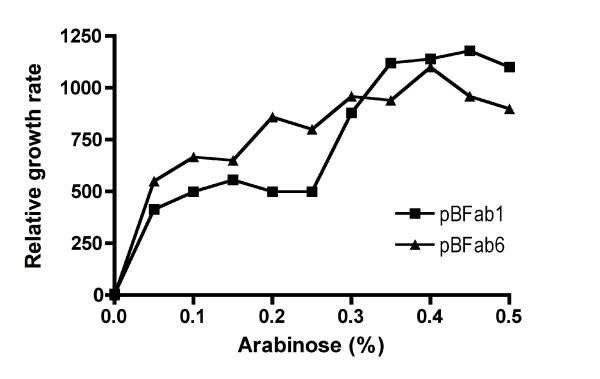
**Effect of *fabI *induction on triclosan resistance**. Triclosan resistance mediated by arabinose-induced over-expression of *fabI*. Exponential growth rates are for DH5α/pBFab1 and DH5α/pBFab6 relative to DH5α/pBAD18s and DH5α/pBAD18, respectively.

To confirm that *fabI *expression was the main mechanism mediating triclosan resistance and not point mutations within chromosomal *fabI *[12], expression of *fabI *was placed under the control of the P_BAD _promoter in pBAD18s (pBFab1) and pBAD18 (pBFab6) [31]. The pBFab1 and pBFab6 strains were tested for resistance following *fabI *induction. In the absence of the inducer arabinose, no growth was observed in LBT broth, but growth rates increased with increasing arabinose concentrations up to 0.4% (Figure 2). Therefore, pFab-mediated resistance to triclosan is due to expression of *fabI*.

### Characterization of pFab

**Figure 3:**
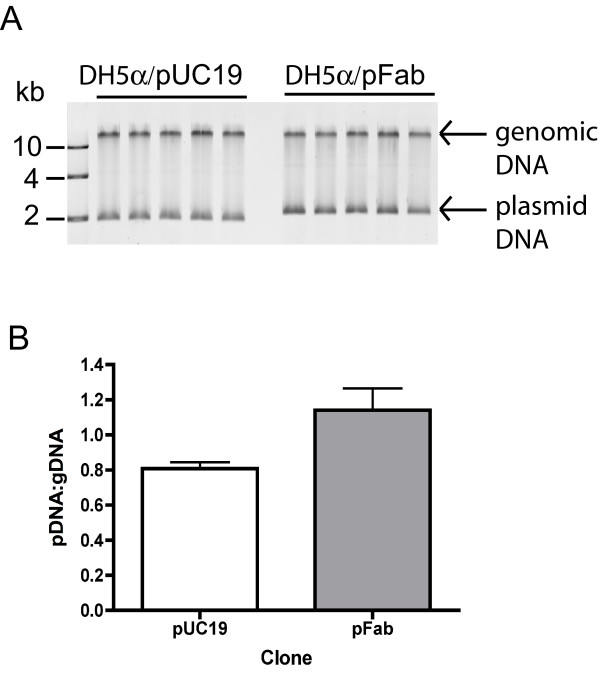
**Plasmid production of pFab transformants**. (**A**) Agarose gel electrophoresis of total DNA isolated from five clones of pUC19 and pFab. Bands were quantified by using ImageQuant software to calculate ratio of pDNA to gDNA as a measure of copy number in pUC19 and pFab. (**B**) Mean ratios of pDNA:gDNA of pUC19 and pFab from (**A**).

After selection of pFab transformants with triclosan, we characterized the general properties of pFab as a cloning vector, with pUC19 included for comparison (Table 1). Plasmid preparation yield of pFab was 43% greater than pUC19 (Table 1) in *E. coli *DH5α transformants. Also, the copy number of pFab was 38% and 40% greater than that of pUC19, as measured by qPCR and plasmid to genomic DNA abundance (Table 1; Figure 3A & B), respectively. Therefore, pFab displayed higher yield and copy number relative to the parent vector.

**Table 1:** Properties of pFab and pUC19

Parameters^a^	pUC19	pFab
Transformation efficiency^b ^(CFU/μg)	5.5 × 10^7 ^± 1.2 × 10^7^	2.2 × 10^7 ^± 3.6 × 10^6^
Plasmid yield^c ^(μg/ml)	30 ± 2.1	42.9 ± 3.5
Copy number^d^	141 ± 25	200 ± 33
Stability (%)		
With selection^e^	85.3 ± 10.8	99.5 ± 1.5
Without selection^f^	56.7 ± 17.7	54.5 ± 14.9
Relative growth rate^g ^(ΔOD/Δt)		
With selection	1	1.2 ± 0.07
Without selection	1.1 ± 0.05	0.6 ± 0.02

^a ^Determined from five replicate cultures.

^b ^In chemically competent DH5α cells.

^c ^Plasmid yield from 1 ml of 18 h culture, determined by OD_260 _absorbance.

^d ^Determined from the copy ratio of *lacZα *to *dxs *by qPCR.

^e ^Percentage of plasmid-bearing cells at 48 h in cultures grown with selection.

^f ^Percentage of plasmid-bearing cells at 48 h in cultures grown without selection.

^g ^Change in OD_550 _over time of DH5α/pUC19 cultures in LB and DH5α/pFab in LB or LBT, relative to control DH5α/pUC19 cultures in LBA.

To assess plasmid stability, we first scored the number of triclosan resistant colony forming units relative to total colony forming units. Surprisingly, we observed more colonies on LBT than on LB plates. This indicated high plasmid stability in the presence of triclosan, but some form of pFab-mediated toxicity in the absence of triclosan. Indeed, measurements of plasmid stability using an alternative α-complementation assay revealed that pFab was more stable than pUC19 under selection (Table 1). Therefore, while pFab over-expression clearly conferred triclosan resistance, it also appeared to confer a requirement for the biocide.

### Effects of pFab and triclosan on *E. coli *growth, survival and fitness

**Figure 4:**
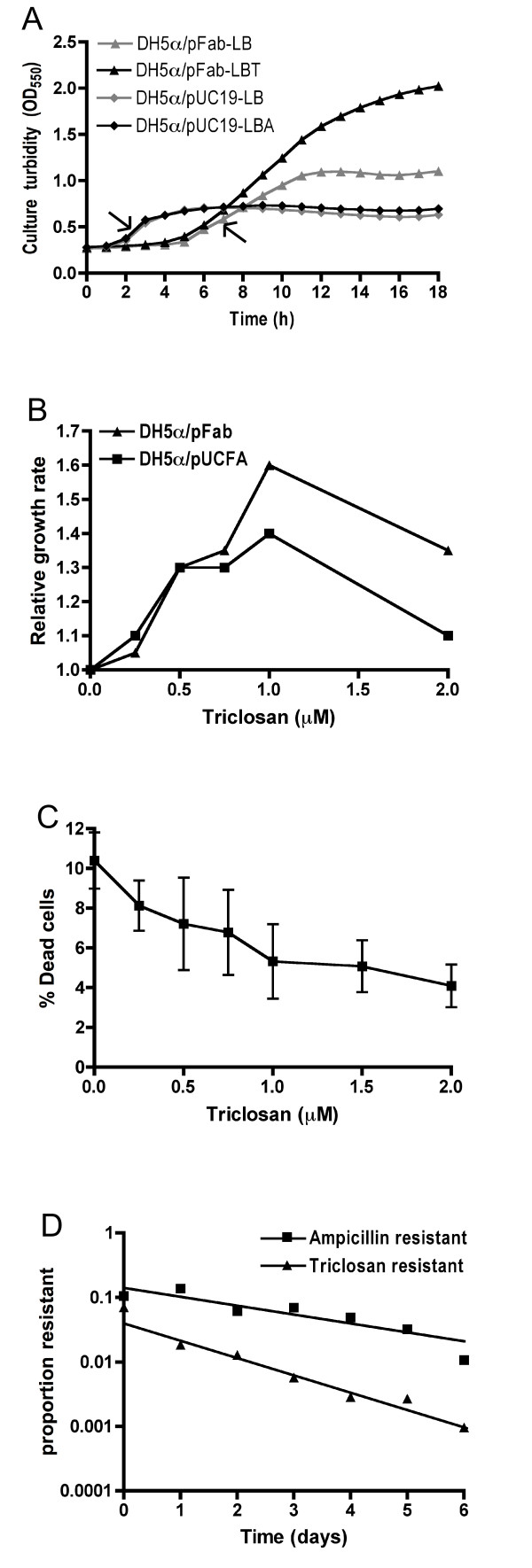
**Effect of triclosan on growth, viability and fitness pFab transformants**. (**A**) Growth curves for DH5α/pUC19 and DH5α/pFab cultures grown with and without selection. Growth rates were determined from the exponential phase, indicated by arrows. (**B**) Reduced fitness with *fabI *over-expression and its suppression by triclosan. Growth rates of pFab and pUCFA clones in LB and LBA, respectively, were calculated relative to DH5α/pUC19 in LBA. (**C**) Flow cytometric determination of dead cell numbers, as a measure of cell toxicity, in DH5α/pFab cultures grown in the presence of a range of triclosan concentrations. (**D**) Growth competition between DH5α and DH5α/pUC19 or DH5α/pFab. The log10 ratio of plasmid-bearing cells to total number of cells against time represents the rate of plasmid loss in mixed cell populations. The data is representative of two independent experiments.

To further investigate the interaction between *fabI *and triclosan, we first examined culture growth profiles. While ampicillin had little effect on DH5α/pUC19 cultures, the final optical density of DH5α/pFab was highest in the presence of triclosan and the exponential phase growth rates of DH5α/pFab cultures were faster than DH5α/pUC19 cultures under selection, whereas the inverse was observed without selection (Figure 4A; Table 1). We next determined DH5α/pFab culture growth rates in the presence of a range of triclosan concentrations (0 – 2 μM). As pFab was unstable without triclosan, DH5α/pUCFA grown in LBA was included to prevent the growth of competing plasmid-free cells. Growth rates of pUCFA and pFab clones were lowest without triclosan and increased with triclosan addition up to 1 μM, indicating a triclosan-dependent fitness rescue in cells that carry pFab (Figure 4B). Similarly, we observed that the proportion of dead cells decreased with increasing triclosan concentrations. Fluorescence microscopy of the SYTOX stained cells [29] revealed elongated and dead cells in the absence of triclosan, and elongated and viable cells in 1 μM triclosan (data not shown). The ratio of live to dead cells indicated by SYTOX staining was then quantified by flow cytometry [29]. The results again showed that triclosan reduced the number of dead cells, indicating that pFab transformants were rescued by triclosan (Figure 4C). Therefore, pFab is toxic but this toxicity is suppressed by triclosan.

To test whether triclosan resistance is likely to persist outside intended applications, we assessed the fitness of DH5/pUC19 and DH5/pFab strains in mixed culture with the plasmid-free parent strain. A competitive fitness assay, conducted in the absence of selection, showed that pFab persistence was weaker than that of pUC19 during co-culture over six days (Figure 4D). In other words, the rate of pFab loss was faster than pUC19 loss and therefore, in the absence of selection, pFab is less stable and less competitive than pUC19.

## Discussion

We describe over-expression of a growth essential gene conferring resistance to a specific protein inhibitor as a plasmid selection system in *E. coli*, using *fabI *and triclosan as an example. As well as avoiding the use of antibiotic resistance genes and antibiotics, pFab transformants showed improved growth, yield and gene containment. These improvements appear to be due to the mechanism of inhibition [13] and a balance of toxic gene-inhibitor levels required for cell survival [32] and selection [33]. Similar interactions in other systems suggest that gene-inhibitor reciprocal suppression may be a common mechanism. For example, reciprocal or mutual suppression has been described between two genes [34] and chemical inhibitors [35]. Also, antibiotic-dependent strains have been described for bacteria isolated from laboratories [36,37] and clinics [38].

The new selection system relies on an endogenous *E. coli *gene and a widely used biocide. Nevertheless, the relative safety of triclosan and *fabI *in this application should be considered. Triclosan is approved by regulatory authorities in the EU and the USA for many applications [24,25] and an association between bacterial triclosan resistance and antibiotic susceptibility, though suggested, has not been found in practice [21-23,39,40]. In the laboratory, spontaneous triclosan resistance in *E. coli *resulting from exposure to low triclosan concentration has been observed, where three point mutations in *fabI *increased MIC by up to 95 fold [12]. Such mutations may arise during triclosan selection. However, in our experiments, triclosan resistance was dependent on expression of plasmid-borne *fabI *(Figure 2), and blue-white selection of recombinant *E. coli *indicated mostly pFab-carrying cells (blue) and very few spontaneous resistant mutants (Table 1). In the environment, triclosan resistance has been slow to emerge compared to antibiotic resistance [21,23], possibly due to poor solubility of triclosan [41], rapid degradation of triclosan [22,42,43], low competitive fitness of FabI mutants [12], and the tripartite nature of the FabI/NADPH/triclosan complex. Furthermore, the spread of pFab outside of intended applications should be limited due to low plasmid stability, poor competitive fitness and cell toxicity in the absence of triclosan. Indeed, FabI is stringently regulated within the fatty acid biosynthesis pathway [32], and de-regulation inhibits cell growth and viability [44,45]. Nevertheless, it is possible that pFab could transfer horizontally and thus induce resistance to triclosan in wild-type bacteria, and standard precautions in the handling of genetically modified microorganisms should be maintained.

In large scale production of proteins, plasmid stability without selection is a pre-requisite because residual antibiotics are undesirable. In this regard, the finding that pFab requires triclosan for plasmid stability could be a disadvantage. However, the amount of triclosan required is much less compared to that in antibiotic selection (typically 2%), and a level of residual triclosan may be permissible, given its approval for use in non-prescription medicines and hygiene products. Therefore, the need for stability in the absence of selection may prove less important with this system. Nevertheless, pFab stability is a potential problem and vector construct or process adjustments may be needed during scale-up.

## Conclusion

In summary, this study provides an example of how essential genes can be used in combination with non-antibiotic inhibitors to select and maintain recombinant bacteria. It may be possible to apply triclosan selection to other bacteria such as *Staphylococcus aureus *[46] and *Mycobacterium smegmatis *[47], which have homologues of FabI. Other inhibitors of FabI have been described [48,49] and, depending on bacterial resistance development, certain triclosan analogues may be preferred for use in this plasmid selection system. On the other hand, bacteria that have divergent enoyl-ACP reductase, such as *Bacillus subtilis *(FabL) [50] and *Streptococcus pneumoniae *(FabK) [51], and species that are intrinsically resistant to triclosan, such as *Pseudomonas aeruginosa *[18], are not suitable for *fabI*-triclosan plasmid selection. For such species, it would be interesting to test additional essential gene inhibitor combinations to expand the plasmid selection strategy described here. The pFab and triclosan system is potentially attractive for production of recombinant proteins, because it can be used to increase plasmid copy number and yield. In addition, pFab and its derivatives may be suitable for manufacturing biopharmaceuticals and gene therapy products, and in other applications that require an absence of antibiotic resistance sequences or antibiotic residues.

## Authors' contributions

SG carried out all experimental work and data analyses except for flow cytometry, participated in design of study and drafted the manuscript. LG conceived the study, carried out flow cytometry and FACS analysis, and helped with design and drafting the manuscript. All authors have read and approved the final manuscript.
